# Hipertensão Arterial Pulmonar: Intervenções para Cardioproteção

**DOI:** 10.36660/abc.20240445

**Published:** 2024-09-04

**Authors:** Elida Paula Benquique Ojopi, Carolina Rodrigues Tonon, Katashi Okoshi, Marina Politi Okoshi

**Affiliations:** 1 Hospital das Clínicas Faculdade de Medicina de Botucatu UNESP Botucatu SP Brasil Hospital das Clínicas da Faculdade de Medicina de Botucatu - Universidade Estadual Paulista (UNESP), Botucatu, SP – Brasil; 2 Faculdade de Medicina de Botucatu UNESP Botucatu SP Brasil Faculdade de Medicina de Botucatu - Universidade Estadual Paulista (UNESP), Botucatu, SP – Brasil

**Keywords:** Hipertensão Arterial Pulmonar, Disfunção Ventricular Direita, Antioxidantes, Homeostase

A vasculatura pulmonar normal é constituída por um sistema de baixa pressão em comparação à vasculatura sistêmica. A hipertensão pulmonar é caracterizada por pressão média na artéria pulmonar acima de 25 mmHg. O processo fisiopatológico primário da hipertensão pulmonar é a restrição ao fluxo sanguíneo na circulação pulmonar, o que leva ao aumento da resistência vascular pulmonar e, eventualmente, à insuficiência ventricular direita. A hipertensão pulmonar pode ser idiopática (HAP) ou resultante de insuficiência cardíaca esquerda, doenças vasculares pulmonares e doença tromboembólica.^1^

Alterações patológicas da estrutura e função dos ventrículos caracterizam a remodelação cardíaca, que é associada a prognóstico desfavorável na HAP.^2^ Atualmente, não há terapia específica para o tratamento da remodelação cardíaca induzida por HAP.^3^

Vários mecanismos estão envolvidos na remodelação cardíaca, tais como estresse oxidativo e alterações do trânsito intracelular de cálcio.^4^ O estresse oxidativo é caracterizado pelo aumento de espécies reativas de oxigênio e nitrogênio.^5^ Importantes fontes de radicais livres são as mitocôndrias e os peroxissomos, que oxidam grupos sulfidrilas pela ação da xantina oxidase produzindo peróxido de hidrogênio e superóxido. Entre os mecanismos de defesa contra as espécies reativas, destaca-se a ação da superóxido dismutase, catalase e glutationa peroxidase que são reguladas, pelo menos parcialmente, pelo fator 2 relacionado ao fator nuclear eritróide 2. Esse fator promove a transcrição de genes relacionados à ação antioxidante.^6^ A contração e relaxamento do músculo cardíaco dependem de adequado trânsito intracelular de cálcio, que é modulado, principalmente, pela cálcio-ATPase do retículo sarcoplasmático (SERCA) e sua proteína regulatória fosfolambam.^7^

A suplementação de compostos bioativos tem sido utilizada para atenuar mecanismos envolvidos na remodelação cardíaca. O suco de uva é rico em flavonoides com propriedades antioxidantes.^8^ A suplementação de hormônios tireoidianos (HT) tem sido avaliada em modelos experimentais de insuficiência cardíaca, tendo disso observada redução do estresse oxidativo e melhora da função cardíaca no infarto do miocárdio experimental. O HT também modula a ativação do Nfr2 promovendo ação antioxidante.^9^

Considerando os possíveis efeitos do suco de uva e da suplementação de HT, foi interessante observar seus efeitos combinados em ratos com HAP induzida por monocrotalina. Em estudo publicado neste volume dos Arquivos Brasileiros de Cardiologia, Proença et al.^10^trataram ratos com HAP com suco de uva e/ou HT via gavagem. Como esperado, os animais com HAP tiveram disfunção sistólica e diastólica do ventrículo direito. A HAP piorou os marcadores de estresse oxidativo xantina oxidase e proteína de choque térmico, a atividade da glutationa peroxidase, e a expressão da proteína SERCA no ventrículo direito. A disfunção sistólica foi atenuada com suco de uva, HT ou terapia combinada, e a disfunção diastólica melhorou somente com HT. A terapia combinada modulou favoravelmente o estresse oxidativo e as variáveis relacionadas ao trânsito intracelular de cálcio.

Os autores concluíram que o suco de uva e os HT, isoladamente ou em combinação, melhoram a função do ventrículo direito em ratos com HAP induzida por monocrotalina. Entretanto, medidas indiretas obtidas por ecocardiografia sugeriram que, usados isoladamente, o suco de uva ou HT reduziram a pressão arterial pulmonar. A redução da hipertensão pulmonar leva a melhora funcional do ventrículo direito, independentemente de alterações na contratilidade miocárdica. Uma limitação do trabalho é o fato que a manobra estressante gavagem não foi realizada em todos os grupos.

O estudo mostrou potenciais benefícios da administração de suco de uva e/ou hormônios tireoideanos na função do ventrículo direito, estresse oxidativo miocárdico e trânsito intracelular de cálcio em ratos com HAP induzida por monocrotalina. Estudos adicionais são necessários para elucidar os efeitos isolados desses compostos na hipertensão pulmonar e inotropismo do ventrículo direito.
