# Supplementary material for: Hipertensão Arterial Pulmonar: Intervenções para Cardioproteção
Source: Arq Bras Cardiol. 2024 Sep 4;121(7):e20240445. [Article in Portuguese] doi: 10.36660/abc.20240445 (PMC11495815; doi:10.36660/abc.20240445)
Supplement: Translation

# Translation: Pulmonary Arterial Hypertension and Cardioprotective Interventions

**DOI:** 10.36660/abc.20240445i

**Authors:** Elida Paula Benquique Ojopi, Carolina Rodrigues Tonon, Katashi Okoshi, Marina Politi Okoshi

**Affiliations:** 1 Brazil Hospital das Clínicas da Faculdade de Medicina de Botucatu - Universidade Estadual Paulista (UNESP), Botucatu, SP – Brazil; 2 Brazil Faculdade de Medicina de Botucatu - Universidade Estadual Paulista (UNESP), Botucatu, SP – Brazil

**Keywords:** Pulmonary Arterial Hypertension, Right Ventricular Dysfunction, Antioxidants, Homeostasis

The normal pulmonary vasculature is a low-pressure system compared with the systemic vasculature. Pulmonary hypertension is characterized by a mean pulmonary artery pressure higher than 25 mmHg. The primary pathophysiological process of pulmonary hypertension is a restriction of blood flow through the pulmonary circulation, which leads to increased pulmonary vascular resistance and eventual failure of the right ventricle. Pulmonary hypertension may be idiopathic (PAH) or aconsequence of chronic diseases, including left-sided heart failure, parenchymal lung diseases, and thromboembolic disease.^1^

Pathological structural and functional changes of the ventricles are known as cardiac remodeling, which is associated with a poor prognosis in PAH.^2^ Currently, no specific treatment is recommended for PAH-induced cardiac remodeling.^3^

Several mechanisms are involved in the pathophysiology of cardiac remodeling, such as oxidative stress and intracellular calcium transient changes.^4^ Oxidative stress is characterized by an increase in reactive oxygen and nitrogen specie.^5^ The main cell sources of free radicals are the mitochondria and peroxisomes, which oxidate sulfhydryl groups by the action of xanthine oxidase producing hydrogen peroxide and superoxide. Important components of the antioxidant system are superoxide dismutase, catalase, and glutathione peroxidase, which are regulated, at least partially, by the nuclear factor erythroid 2–related factor 2 that promotes the transcription of antioxidant genes.^6^ Cardiac muscle contraction and relaxation depend on adequate intracellular calcium handling, which is mainly controlled by the sarcoplasmic reticulum calcium-ATPase (SERCA) and its regulatory protein phospholamban.^7^

Supplementation of bioactive compounds has been used to attenuate mechanisms involved in cardiac remodeling. Grape juice is a rich source of flavonoids with antioxidant properties.^8^ Thyroid hormone (TH) supplementation has been tested in heart failure models. It decreased oxidative stress and improved cardiac functional parameters in experimental myocardial infarction. TH also modulates Nfr2 activation, promoting an antioxidant environment.^9^

It was interesting to observe the effects of combined grape juice and TH administration in rats with monocrotaline-induced PAH. In a study published in this issue of *Arquivos Brasileiros de Cardiologia*, Proença et al.^10^treated PAH rats with grape juice and THs via gavage. As expected, monocrotaline-treated rats had right ventricular systolic and diastolic dysfunction. The oxidative stress markers xanthine oxidase and 70kDa heat shock protein, glutathione peroxidase activity, and expression of SERCA were impaired in the right ventricle of the PAH rats. Systolic dysfunction was attenuated with grape juice, TH, or the combined therapy, and diastolic function improved with TH alone. Both treatments improved oxidative stress and intracellular calcium transient-related parameters.

The authors conclude that grape juice and TH, alone or combined, improve right ventricular functional parameters in rats with monocrotaline-induced PAH. However, indirect measurements obtained by transthoracic echocardiogram suggest that isolated grape juice or TH reduced pulmonary artery pressure. By decreasing pulmonary hypertension, right ventricular function improves independently of contractility changes. A limitation of this study is the fact that the stressful gavage was not performed in all groups.

The study highlighted the potential benefits of grape juice and/or TH administration in right ventricular dysfunction, myocardial oxidative stress, and intracellular calcium transient in rats with monocrotaline-induced pulmonary artery hypertension. Additional studies are needed to elucidate the isolated effects of these compounds on pulmonary hypertension and right ventricular inotropism.
